# Medication Utilisation Program, Quality Improvement and Research Pharmacist—Implementation Strategies and Preliminary Findings

**DOI:** 10.3390/pharmacy9040182

**Published:** 2021-11-04

**Authors:** Karen Whitfield, Ian Coombes, Charles Denaro, Peter Donovan

**Affiliations:** 1 Department of Clinical Pharmacology, Royal Brisbane and Women’s Hospital, Butterfield Street Herston, Brisbane, QLD 4029, Australia; peter.donovan@health.qld.gov.au; 2 School of Pharmacy, University of Queensland, 20 Cornwall Street, Brisbane, QLD 4102, Australia; ian.coombes@health.qld.gov.au; 3 Department of Pharmacy, Royal Brisbane and Women’s Hospital, Butterfield Street Herston, Brisbane, QLD 4029, Australia; 4 Department of Internal Medicine & Aged Care, Royal Brisbane and Women’s Hospital, Butterfield Street Herston, Brisbane, QLD 4029, Australia; c.denaro@uq.edu.au; 5 Faculty of Medicine, University of Queensland, 288 Herston Road, Herston, QLD 4006, Australia

**Keywords:** medication utilisation program, medication optimisation, pharmacist, clinical pharmacology, medication quality improvement, medication research, medication-use evaluation

## Abstract

Judicious use of medicines that considers evidence-based practice, together with cost-effectiveness, is a priority for all health care organisations. We describe an initiative to lead a Medication Utilisation Program, incorporating medication quality improvement and research activities. In August 2020 an advanced pharmacist position was implemented to lead the Program. The purpose was to provide oversight and facilitate initiatives promoting medication optimisation to create sustainable change in practice. A strategic plan was developed with key performance indicators. A governance structure was implemented with relevant reporting mechanisms. Strategic planning and collaboration with medical, nursing and allied health professionals has seen the successful implementation of seven codesigned medication-use evaluations and eight quality improvement projects centred around patient safety, quality and value-based care. Several research studies have been designed with subsequent commencement of pharmacists enrolled in university Research Higher Degree programs. Cost containment initiatives have realised potential savings approximating AUD 250,000. Educational programs included protocol design, ethics approvals and report writing. Key success criteria for a Medication Utilisation Program include dedicated pharmacist resources, structured governance and reporting mechanisms. Alignment of study complexity with staff experience and interdisciplinary collaboration are also critical.

## 1. Introduction

Quality use of medications promotes judicious, appropriate and safe use of medicines. Judicious use of medicines can occur through considering the place of medicines in treating illness and maintaining health, and by recognising that there may be better ways than medicine to manage many disorders [1,2].

*Crossing the Quality Chasm* by the Institute of Medicine outlined specific aims that a healthcare system must fulfill to deliver quality care and are useful to take into account when considering quality use of medications [3]:

- Safe, avoiding injuries to patients from the care that is intended to help them.
- Effective, providing services based on scientific knowledge to all who could benefit and refraining from providing services to those not likely to benefit (avoiding underuse and overuse, respectively).
- Patient-centred, providing care that is respectful of and responsive to individual patient preferences, needs, and values and ensuring that patient values guide all clinical decisions.
- Timely, reducing waits and sometimes harmful delays for both those who receive and those who give care.
- Efficient, avoiding waste, including waste of equipment, supplies, ideas and energy.
- Equitable, providing care that does not vary in quality because of personal characteristics such as gender, ethnicity, geographic location and socioeconomic status.

There are several ways to ensure optimisation and quality use of medications, and these include medication-use evaluations, quality improvement initiatives and research studies [4]. The American Society of Health-System Pharmacists (ASHP) in their 2021 guideline on medication-use evaluation describe the essential elements of the process for health care organisations [5]. A systematic and interdisciplinary approach to optimise patient outcomes using ongoing evaluation to improve medication utilisation is the main goal. A recommendation is made to identify the focus of the medication-use evaluation, and this may be patient-centred therapeutic outcomes, but also may be parts of the process, for example, prescribing, administration or communication. These recommendations are similar to those described by Fanikos et al. (2014) that suggested medication-use evaluations (MUE) be utilised for three situations [6]:

- When the benefit of the medication is unknown;
- When there are little data available to influence choice;
- When there is a need to analyse the process of medication management including prescribing, preparation, dispensing, administration and monitoring.

Problems and barriers were identified in the ASHP guideline [5] with an explanation how to address, that included the need for:

- Authoritative medical staff support;
- Clear structure and leadership with clear definition of roles, responsibilities and accountabilities;
- Excellent communication and the importance of involvement of key stakeholders;
- Excellent documentation summarising findings and outlining an action plan;
- An interdisciplinary approach;
- Follow up and evaluation of initial actions, flexibility to adjust the action plan if necessary and keeping sight of improvement goals;
- Education regarding not only how to conduct these studies but also education when implementing new processes.

Owing to limited resources and financial constraints within many hospital organisations it is imperative to prioritise MUEs. These may be related to patient-specific outcomes, such as safety, medication effectiveness, appropriate dosing, quality standards and cost [6,7]. 

Medication-use evaluations can form a part of a larger quality improvement initiative. Quality improvement aims to make a difference to patients by improving safety, effectiveness, and experience of care by utilising a systematic approach and designing, testing and implementing changes using real time measurement for improvement [8]. Defining the difference between quality improvement and research is challenging and they can overlap. Quality improvement focuses on systems and processes and uses measures to determine if a change has led to improvement, whereas research will formulate an hypothesis, systematically study the issue of interest, collect and analyse results and disseminate the findings regardless of a positive or negative find [9].

The aim of this work is to describe the implementation and preliminary findings of a pharmacist specifically responsible for the oversight of a Medication Utilisation Program, incorporating medication-use evaluations, quality improvement projects and research studies. The work will also outline strategies put into place for success, including strategic planning, governance and reporting structures. 

## 2. Materials and Methods

The concept of the Medication Utilisation Program (MUP) pharmacist position started in February 2020 following consultation with the Director of Clinical Pharmacology and Director of Pharmacy at a tertiary teaching hospital in Queensland Australia. A gap was identified for an advanced pharmacist to lead a Medication Utilisation Program that incorporated oversight of medication related studies. Role establishment, purpose and governance over a 12-month period are described below.

### 2.1. Establishment of the Role

The Medication Utilisation Program pharmacist was established in August 2020. The role reports directly to the Director of Clinical Pharmacology with a professional reporting line to the Director of Pharmacy. The MUP pharmacist works directly with the Clinical Pharmacology Department and the Pharmacy Department with a vision to lead and facilitate initiatives promoting medication optimisation across the hospital, to create a sustainable change in practice.

### 2.2. Purpose of the Role

**Figure 1:**
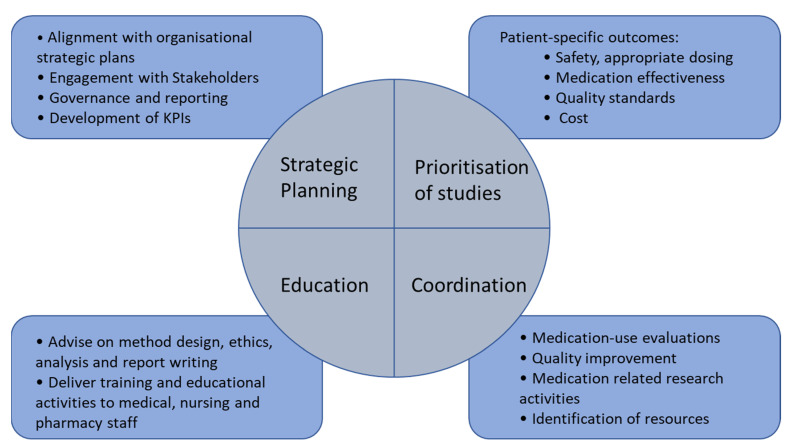
The role of the MUP pharmacist.

The roles of the MUP pharmacist are concluded in Figure 1.

- To lead the strategic planning and implementation of a Medication Utilisation Program to include medication quality improvement and medication related research activities.
- To coordinate medication-use evaluations, quality improvement and medication related research activities including: the evidence-based review of medicines use, review of medication expenditure, and the implementation and evaluation of interventions to change practice in collaboration with medical, pharmacy and nursing staff across all service lines of the hospital.
- To apply, implement and evaluate the Medication Utilisation Program in cost-effectiveness and patient outcomes, in alignment with the Australian Commission’s National Safety and Quality Health Service Standards.
- To implement the Medication Utilisation Program with a focus on high cost, high usage and high-risk medications to ensure cost-effective, evidence-based medication use is implemented to optimise patient outcomes.
- To develop and deliver training and educational activities associated with medication utilisation review, quality improvement and research activities to medical, nursing and pharmacy staff.

### 2.3. Governance Structure

The activities of the MUP pharmacist are governed by the Quality Use of Medicines (QUM) Subcommittee which in turn reports to the Hospital Medicines Advisory Committee. The overall purpose of the QUM Subcommittee is to coordinate the organisational response for the management of QUM in accordance with best practice. Through its activities, this Subcommittee aims to ensure the implementation, sustainability and ongoing improvement of practices related to medications across the hospital. One of the main responsibilities of the committee is to guide the implementation of strategies to improve QUM within the organisation to reduce patient risk. In particular, this includes support strategies which improve governance and management of high-risk or low-value medications. The membership includes representation from disciplines and specialities from across the hospital and is chaired by the Director of Internal Medicine and Aged Care.

### 2.4. Cost Containment Activities

A record of all cost containment activities was kept. Cost savings were calculated depending on activity. Savings generated by changes to preferential contracts, improvement in stock management, and correct reimsbursment, for example, were made on 12-month usage.

### 2.5. Dissemination of Study Findings

Dissemination of Medication Utilisation Program study findings and activities were reported and made available to all relevant stakeholders, including the Director of Clinical Pharmacology, Director of Pharmacy, the High-Cost Drugs Committee, and the Medicines Advisory Committee. All staff undertaking these activities are encouraged to submit manuscripts for publication to peer reviewed journals and submit abstracts for conference presentation.

## 3. Results

### 3.1. Strategic Planning

The Medication Utilisation Program strategic plan (Table 1) was developed to align with the hospital’s strategic plan for 2020–2024 that includes objectives, strategies and key performance indicators (KPIs). KPIs were created using several principles, including ensuring that they were easy to measure, feasible and clearly reflecting progress and performance. SMART (specific, measurable, achievable, realistic and time bound) objectives were used in the development. KPIs will be reviewed after 18–24 months post implementation of the role. 

Engagement with service line Medical and Nursing Directors including Medicine, Surgery, Cancer Care, Cardiology, Renal, Women’s and Newborns and Pharmacy was undertaken to explain the MUP pharmacist role and discuss potential ideas for medication-use evaluations (MUEs), quality improvement and research studies.

### 3.2. Development of MUP Studies

Table 2 outlines MUEs that were prioritised to be undertaken as a result of high-cost medications, anecdotal evidence for nonadherence to guidelines and/or hospital formulary restrictions. MUEs were led by pharmacist interns (preregistration pharmacists) under the direct supervision of a senior pharmacist. The information in Table 2 serves to give a brief overview of the MUEs and outcomes to date only. The MUP pharmacist aligned all MUEs with the MUP strategic plan objectives. 

Table 3 outlines medication related quality improvement studies that were proposed via several mechanisms. These included studies identified by department or service line Directors, pharmacy staff, as well as follow up from previous work. Many of the studies are being led by resident pharmacists or senior pharmacists. The information in Table 3 serves to give a brief overview of the quality improvement study, the rationale for conducting and potential outcomes only. These studies are planned to take 12–18 months and are all in progress. The MUP pharmacist aligned all quality improvement studies with the MUP strategic plan objectives.

The MUP pharmacist had oversight of all MUEs and quality improvement projects. MUP pharmacist involvement included ensuring robust protocol design, advice of appropriate ethics applications, support during analysis of the results, and review and editing of the final report. 

Table 4 outlines some of the research studies that are in progress. All research studies outlined in Table 4 are being undertaken by senior pharmacists. Their supervisory teams are all multidisciplinary and include a senior physician and, in some cases, an academic nurse. All students are enrolled in a Research Higher Degree with an academic institution. The MUP pharmacist is the primary research supervisor of six out of the seven studies. The MUP pharmacist aligned all research studies with the MUP strategic plan objectives.

Table 5 outlines the KPIs that have been achieved and those that have not been achieved over the 12 months, post implementation of the role.

### 3.3. Cost Containment Activities

Several cost containment activities were implemented during the 12 months in collaboration with pharmacists, pharmacy purchasing and support staff. These included reduction in wastage, improvement in stock management, greater adherence to local medication formularies, raising awareness of high-cost medications with pharmacy staff and appropriate claiming of reimbursement. Costs savings amounted to approximately AUD 250,000.

### 3.4. Educational Activities

Training and education sessions developed and delivered included how to conduct a medication-use evaluation, introduction to quality improvement frameworks including the Plan-Do-Study-Act (PDSA) models, how to navigate and undertake suitable ethics processes, how to present results to multidisciplinary audiences and how to write a report.

## 4. Discussion

In 2020, a new initiative to implement a Medication Utilisation Program pharmacist to lead and oversee medication-use evaluations, medication quality improvement and research activities was undertaken at a tertiary teaching hospital. The importance of developing a strategic plan that aligned with the organisation’s strategy and key objectives cannot be underestimated. The benefits of this not only relate to projects that are well designed, add value to the organisation, focus on quality of care given to patients and cost-effectiveness, but also allows evaluation of how the Program is performing against key performance indicators. Several of the key performance indicators have been achieved such as conducing a set amount of MUEs, demonstrating cost-effective initiatives, demonstrating staff participation in MUP activities and conducting educational activities. Areas to improve and focus on include initiatives that involve consumers and MUEs related to high-risk medications. 

It was useful to reflect on the problems and pitfalls to avoid that were outlined in the recently updated ASHP guidelines on medication-use evaluation and ensure that factors such as having medical leadership and support, taking an interdisciplinary approach and excellent communication were in place [5]. Ensuring governance structures were in place was key for transparency of work undertaken, as well as an enabler to decide prioritisation of projects that align with organisational goals. Regular reporting to high level committees such as the Quality Use of Medicines Subcommittee and the Hospital Medication Advisory Committee provide a communication pathway to Medical and Nursing Directors to disseminate findings and outcome of studies. 

In any successful program identifying staffing resources to undertake projects is essential. Resources included final year pharmacy students undertaking an honours elective or quality use of medicine placement, pharmacist interns, resident pharmacists and senior pharmacists. Complexity of studies together with time frame were taken into consideration when identifying suitable people to take the lead on studies. The formalisation of pharmacist advanced development is a global opportunity for pharmacists to develop nonclinical and pharmacy distribution roles and upskill in evaluation of medication management by undertaking audits, medication-use evaluations and more structured research projects [10,11]. The formalised advanced training programs also facilitate clinical networking and collaboration and research and presentation and publication skills, all of which are essential for medication-use evaluations. 

Almost all the medication-use evaluations, quality improvement and research studies were designed and/or undertaken in collaboration with specialist medical, nursing or allied health professionals in the field. Excellent examples of these include MUEs undertaken on high-cost antimicrobial agents that included the Antimicrobial Stewardship team, and MUEs on opioid use involving the acute pain team. Examples of collaboration in quality improvement studies include speech pathologists looking at effective medication management of patients with dysphagia, and staff specialists in neonatology looking at effective and safe antihypertensive agents in critically ill infants. There is substantial evidence that a multidisciplinary approach to delivering health care is essential to delivery of best patient outcomes [12,13]. The same approach should be considered when undertaking quality improvement or research studies, to ensure robust design as well as effective implementation and evaluation. In addition, key components of effective quality improvement programs need to include education, ideally by an individual or team with an educational focus to disseminate the information regarding the intervention [14].

Evidence suggests that despite organisations continuing to build strategies to optimise medications, there are challenges to effectively demonstrating impact on cost and quality outcomes [2]. Important factors identified for success include integration of pharmacists into care teams, access to clinical data by pharmacists and physicians, physician buy-in and measuring impact of initiatives. The introduction of the MUP pharmacist to professionally lead project design development has seen the development and implementation of projects with a robust framework that have the potential to demonstrate impact on cost and quality. As outlined in the ASHP guidelines it is important to follow up, evaluate initial actions, be flexible to adjust the action plan if necessary and keep sight of improvement goals in order to demonstrate impact on cost and quality outcomes [5].

Several cost containment activities have been implemented since the MUP pharmacist started and these generally involved reduction in wastage, stock management, appropriate claiming of reimbursement and raising awareness of high-cost medications within the department. These types of cost saving initiatives are important, as outlined in the *Crossing the Quality Chasm* report, and more than offset the cost of the pharmacist [3]. It is also important to consider economic analysis of MUEs and quality improvement initiatives, which can be more challenging. Examples where this has been incorporated as part of the analysis in quality improvement studies relatively simply include intravenous to oral conversions [15]. Examples where this is more challenging include studies involving the impact of medication nonadherence [16].

Several pharmacists within the department have enrolled in Research Higher Degrees and are undertaking research that will be translated directly into practice. Studies include topical and relevant subjects such as exploring the benefits of early discharge follow up for high-risk patients by pharmacists as a strategy to mitigate the burden of nonadherence to medications, and risks associated with continuing care across the continuum into primary care. Other research studies involve exploring ways to improve care delivered to patients, identifying value-based care and cost-effective strategies. All students have either a multidisciplinary supervisory team or an expert invited to join the team at relevant points during the study. The MUP pharmacist is the principal advisor for six of these students and also holds a conjoint research academic appointment one day a week, which is a key enabler to be involved with and advise Research Higher Degrees students.

Several training and education sessions were developed and delivered. These mainly involved how to conduct a medication-use evaluation, using frameworks including the PDSA models, how to navigate and undertake suitable ethics processes, as well as writing a report. These sessions were predominantly delivered to pharmacy staff, and it is hoped that these could be extended to include the wider multidisciplinary team of medical, nursing and other allied health professionals. Future training sessions will incorporate how to make time in the working day to conduct projects, as this was highlighted as a barrier by several individuals. 

### 4.1. Limitations

The authors would like to outline some limitations. This position was implemented within the setting of a tertiary teaching hospital, for which resources were allocated. Such positions may not be feasible in smaller organisations and those where pharmacy resources are limited. However, the strategies employed, and the processes used to develop, implement and evaluate the position may be useful to build a future business plan. This initiative is only in the first year and as such further evaluation needs to be undertaken particularly with implementation of more complex quality improvement and research studies. However, results from several studies undertaken together with cost containment initiatives have demonstrated positive outcomes. Finally, it can be seen that many of the studies, although collaborative in nature using the wider multidisciplinary team, are led by pharmacists. The vision for the future is that more medical officers and nursing staff will lead medication related collaborative studies. Time commitments to lead projects may be a barrier for some medical and nursing staff. 

### 4.2. Future Directions

The MUP pharmacist role has been an interesting and exciting role to implement and evaluate progress to date. However, looking forward to the next 12 months there are challenges to overcome. Ensuring the progression and momentum of projects continues is critical, particularly with quality improvement projects that will take 12–18 months to complete. Contingency plans need to be in place, for example when people leading or involved in studies leave the place of work or take other roles that do not allow them to continue with the work. Motivation can also diminish when barriers and unexpected obstacles are encountered. Education and support can assist here to reassure, particularly junior staff, that this is often normal and provides an opportunity to develop critical thinking and problem-solving skills. In addition, it is imperative to build a workforce that is adequately trained and supported to undertake MUEs, quality improvement and research studies. Another challenge to combat is ensuring the sustainability of implemented interventions. Change management theory and concepts are essential to use here, including models such as the ADKAR change management model, Kotter’s theory and McKinsey 7S model [17,18,19,20,21]. Future directions will also include continued dissemination of findings using a range of formats including conference proceedings locally and nationally, as well as increasing peer reviewed publication output.

## 5. Conclusions

Key success criteria for a Medication Utilisation Program include dedicated pharmacist resources, structured governance and reporting mechanisms. Having a dedicated resource to lead and oversee medication related activities has many benefits, including ensuring that studies align with hospital strategic plans, that studies are well designed, add value to the organisation and focus on quality of care. The MUP pharmacist also has the ability to align study complexity with staff experience to improve efficiency and help maintain momentum and motivation. Interdisciplinary collaboration is critical for any successful program and the MUP pharmacist can ensure that this takes place with the relevant staff and stakeholders. 

## Figures and Tables

**Table 1:** Medication Utilisation Program pharmacist strategic plan and KPIs aligned with hospital’s strategic plan.

Objective 1 To Always Put People First.	Objective 2 To Improve Health Equity, Access, Quality, Safety and Health Outcomes.	Objective 3 To Deliver Value-Based Health Services through a Culture of Research, Education, Learning and Innovation.	Objective 4 To be Accountable for Delivery of Sustainable Services, High Performance and Excellent Patient Outcomes.
1.1 Engagement with consumers and caregivers, with MUP initiatives 1.2 Engagement with staff with MUP initiatives 1.3 Engagement with partners with MUP initiatives 1.4 Provide staff with support, education, training and development opportunities related to medication use, evaluation, quality improvement and research initiatives	2.1 Conduct MUP activities to identify any risk and to develop, implement and evaluate strategies to address 2.2 Conduct MUP activities with a focus on high-risk medications including APINCH * medications 2.3 Conduct MUP activities that promote an evidence-based approach to patient care 2.4 Conduct MUP activities that create system capacity through workforce, infrastructure, technology, service development and redesign. 2.5 Conduct MUP activities to monitor and evaluate digital prescribing 2.6 Conduct MUP activities to minimise risk at clinical handover especially on discharge 2.7 Conduct MUP activities to ensure safe and appropriate prescribers	3.1 Implement sustainable initiatives that utilise strategies for cost-effective use of medications 3.2 Conduct MUP activities that improve governance and management of low-value medications to disinvest in low-value healthcare 3.3 Develop strategic collaborations to generate new knowledge through research, evaluating what others have learned and actively bringing this knowledge into practice 3.4 Create an environment that promotes innovative approaches to support staff in continuous improvement and organisational learning 3.5 Collaborate with partners to facilitate clinical placement requirements, associated with MUP projects	4.1 Conduct MUP activities that enable models of care that make most effective use of available and future resources including redirecting investment where evidence supports new or alternative practices 4.2 Regular reporting to relevant Medical and Nursing leads and to the Medications Committee 4.3 Develop robust governance processes over MUP activities that utilise a robust evaluation and impact framework
**Key Performance Indicators**			
1.1 Demonstrate at least three codesigned MUP initiatives with consumers per annum 1.2 Demonstrate staff participation in MUP activities 1.3 Demonstrate MUP educational activities for staff	2.1 Conduct 5 MUEs per year 2.2 Conduct 3 MUEs activities related to high-risk medications per year	3.1 Demonstrate at least three cost-effective initiatives per annum 3.2 Demonstrate involvement in staff pursuing a higher degree	4.1 Achieve sustainable positive financial results. 4.2 Demonstrate projects that have a robust evaluation framework

* APINCH—antimicrobial, potassium and other electrolytes, insulin, narcotics and sedative agents, chemotherapy and heparin and other anticoagulants.

**Table 2:** Medication-use evaluation activities.

MUE and Rationale	Outcome	Objective Alignment
**Poractant Alfa use in Neonatal Intensive Care** High-cost medication and adherence to guidelines	Very high level of adherence to guidelines. Appropriate dosing in all but a small number of cases. Minimal adverse effects. Recommendations made to monitor and improve future prescribing were accepted.	1.2, 2.7, 3.1
**Tapentadol use in admitted patients** Opioid stewardship and adherence to local hospital formulary	High level of adherence to guidelines. Use of multiple agents prescribed for breakthrough pain in 50% of patients. Recommendations to liaise with acute pain team were accepted.	1.2, 2.2, 2.7
**Botulinum Toxin type A use** High-cost medication and adherence to local hospital restrictions	Very high level of adherence to guidelines and hospital approvals. Recommendations made regarding education for medical and pharmacy staff to raise awareness of necessary approvals.	1.2, 3.1
**Intravenous aciclovir** High-cost medication and adherence to local hospital restrictions	Very high level of adherence to guidelines and hospital restrictions. Evidence of appropriate antimicrobial stewardship team involvement. Recommendations to review intravenous aciclovir usage annually were accepted.	1.2, 2.2, 3.1
**Nebulised pentamidine in cancer care for PJP prophylaxis** High-cost medication and appropriate use	A number of patients identified as suitable for Bactrim retrial and direct de-labelling and/or possible for desensitisation. Several recommendations made regarding education to nursing and medical staff to raise awareness of desensitisation guideline and internal referral to de-labelling clinic were accepted. Re-evaluate after implementation of recommendations.	1.2, 3.1
**Melatonin use in admitted adolescents (mental health ward)** Adherence to interim hospital approval in select adolescent patients	Patients prescribed melatonin prior to admission and supply continued. Reduction in use of some sedating medications. Recommendation for further study to investigate medications used to assist sleep in admitted patients for efficacy and adverse effects.	1.2
**Intravenous fluid use** High-cost medications and adherence to local hospital formulary	IV fluid usage overall appeared appropriate. Two non-approved fluids in use. Recommendations made to investigate non-approved fluids and suggest alternatives. Recommendations to update the local prescribing IV fluid guideline for adults were accepted. Re-evaluate after implementation of recommendations.	1.2, 3.1

**Table 3:** Quality improvement Medication Activities.

Study	Rationale	Potential Outcomes	Objective Alignment
Evaluation of the correlation, preventability and severity of hospital acquired complications. specifically hemorrhage from anticoagulant medications	Retrospective study of patients with a hospital acquired complication (HAC) coded in relation to anticoagulation medications. This is a high-risk medication. Adverse effects contribute to poorer clinical outcomes for patients, and often increase length of stay in hospital, increasing financial burden on the health system.	Identification of common underlying causes for hemorrhagic hospital acquired complications from anticoagulants. Identification of the appropriateness of hospital acquired complication (HAC) coding in relation to anticoagulants.	2.1, 2.2
To evaluate the use of LAMA * and LABA ** inhaler therapy following temporary hospital approval for continuation of therapy in admitted patients	LAMA * and LABA ** are expensive inhalers and not currently hospital approved. Interim alternative inhalers are supplied for admitted patients who do not bring in their own inhalers to hospital. This can result in confusion to patients and wastage.	To determine if the implementation of a hospital approval for continuing supply of LAMA * and LABA ** inhalers for admitted patients has resulted in a reduction in cost and wastage associated with interim and or alternative hospital approved inhalers.	3.1
To evaluate pharmacist confidence in documenting recommendations in patient clinical notes in a digital hospital compared to a paper-based hospital	Anecdotal evidence suggests documenting recommendations in the patient clinical notes by pharmacists is inconsistent.	Identification of any differences between pharmacists’ confidence documenting in patient clinical notes, at digital or paper-based site, and if so the barriers and enablers for this.	1.2, 1.3
To examine health care staff communication, knowledge and practices regarding medication management of patients with dysphagia	This is a collaborative study involving pharmacy and speech pathology. The effective management of patients with dysphagia rely on collaboration between the multidisciplinary team. The study will examine the current practice of the multidisciplinary team in the medication management of patients with dysphagia.	Identification of any gaps in staff communication, knowledge and practices regarding medication management of patients with dysphagia and development of interventions to address these.	1.2, 2.1, 2.6
To determine the use and effectiveness of antihypertensive medications currently used in Neonatal Intensive Care	This is a collaborative study looking at current antihypertensive agents used in critically ill infants to manage hypertensive episodes. Limited evidence is available to guide practice.	Identification of effectiveness of current medications to manage hypertension and any associated adverse events.	1.2, 2.1, 2.2, 2.3
To determine anti-arrhythmic medications prescribed in the peri-procedural and post-procedural period to reduce the risk of arrythmia recurrence	This is a collaborative study to explore the range of anti-arrhythmic medications currently prescribed and the monitoring undertaken compared to current local and international guidelines.	Identification if appropriate monitoring of anti-arrhythmic medications is taking place post discharge; and to identify any associated problems and address if required.	1.2, 2.1, 2.3
Therapeutic Drug Monitoring analysis	As an intervention Therapeutic Drug Monitoring has been shown to improve patient responses to important life-sustaining medications and to decrease adverse drug reactions. Therapeutic Drug Monitoring can have positive economic outcomes; however, these are negated if inappropriate and wasteful testing is undertaken.	Identification of the quantity and range of Therapeutic Drug Monitoring performed and evaluate the usefulness of the result when compared with current guidelines. Identification if appropriate dose adjustment made was appropriate and evaluate any waste.	1.2, 2.2, 3.1, 4.1
To develop and evaluate a tool to efficiently and reproducibly track the cost of claimable and non-claimable medication dispensed within the hospital	Tracking costs of non-claimable medicines and reviewing use that falls outside the Pharmaceutical Benefits Scheme (PBS) indication ensures optimal patient care and financial sustainability of medication use.	Development of a tool to allow efficient, timely analysis of non-claimed and claimed high-cost medicines, projection of high-cost medicine expenses and evaluation of cost changes associated with changes in mediation supply.	3.1

* LAMA—long-acting muscarinic antagonist. ** LABA—long-acting beta2-agonist.

**Table 4:** Research related Medication Activities of Research Higher Degree students.

Study	Objective Alignment
Strategies to investigate oral mucositis prevention in patients undergoing haematopoietic stem cell transplantation (HSCT)	1.2, 1.4, 2.2, 3.3
Investigation to evaluate the impact of a pharmacist-led Therapeutic Drug Monitoring optimisation	1.2, 1.4, 2.2, 3.3
Development and implementation of a risk assessment tool of poisoned patients by non-expert clinicians in Emergency Departments	1.2, 1.4, 2.2, 3.3
Investigation of the impact of a pharmacist in the medication management of patients post bariatric surgery	1.1 1.2, 1.4, 3.3
Investigation of the impact of a multidisciplinary team in the medication optimisation of diabetic patient prior to surgery	1.1, 1.2, 2.2, 1.4, 3.3
Investigation of the impact of a pharmacist in early cardiac patient follow up after hospital discharge	1.1, 1.2, 1.4, 3.3
Characteristics of readmitted patients reviewed by a high-risk discharge pharmacist	1.2, 1.4, 3.3

**Table 5:** Key performance indicator progress.

Key Performance Indicator	Comment
1.1 Demonstrate at least three codesigned MUP initiatives with consumers per annum	Not achieved
1.2 Demonstrate staff participation in MUP activities	Achieved
1.3 Demonstrate MUP educational activities for staff	Achieved
2.1 Conduct 5 MUEs per year	Achieved
2.2 Conduct 3 MUEs activities related to high-risk medications per year	Not achieved
3.1 Demonstrate at least three cost-effective initiatives per annum	Achieved
3.2 Demonstrate involvement in staff pursuing a higher degree	Achieved
4.1 Achieve sustainable positive financial results	Achieved
4.2 Demonstrate projects that have a robust evaluation framework	Achieved

## Data Availability

The datasets generated during and/or analysed during the current study are available from the corresponding author on reasonable request.

## References

[1] Australian Government Department of Health (2020). What is Quality Use of Medicines?. https://www1.health.gov.au/internet/main/publishing.nsf/Content/nmp-quality.htm.

[2] Wilks C., Krisle E., Westrich K., Lunner K., Muhlestein D., Dubois R. (2017). Optimization of Medication Use at Accountable Care Organizations. J. Manag. Care Spec. Pharm..

[3] Institute of Medicine Committee on Quality of Health Care in America (2001). Crossing the Quality Chasm: A New Health System for the 21st Century.

[4] Vest T.A., Gazda N.P., Schenkat D.H., Eckel S.F. (2020). Practice-enhancing publications about the medication-use process in 2018. Am. J. Health Syst. Pharm..

[5] Afanasjeva J., Burk M., Cunningham F., Fanikos J., Gabay M., Hayes G., Masters P.L., Rodriguez R., Sinnett M.J. (2021). ASHP Guidelines on Medication-Use Evaluation. Am. J. Health Pharm..

[6] Fanikos J., Jenkins K.L., Piazza G., Connors J., Goldhaber S.Z. (2014). Medication use evaluation: Pharmacist rubric for performance improvement. Pharmacotherapy.

[7] World Health Organization (2003). Drug and Therapeutics Committees: A Practical Guide. https://apps.who.int/iris/handle/10665/68553.

[8] Jones B., Vaux E., Olsson-Brown A. (2019). How to get started in quality improvement. BMJ.

[9] Gregory K.E. (2015). Differentiating Between Research and Quality Improvement. J. Périnat. Neonatal Nurs..

[10] International Pharmacuetical Federation (2019). FIP Global Advanced Development Framework—Supporting the advancement of the profession. https://www.fip.org/file/4331.

[11] Galbraith K., Coombes I., Matthews A., Rowett D., Bader L.R., Bates I. (2017). Advanced pharmacy practice: Aligning national action with global targets. J. Pharm. Pract. Res..

[12] Helou N., Talhouedec D., Zumstein-Shaha M., Zanchi A. (2020). A Multidisciplinary Approach for Improving Quality of Life and Self-Management in Diabetic Kidney Disease: A Crossover Study. J. Clin. Med..

[13] Mieiro D.B., De Oliveira Érica B.C., Da Fonseca R.E.P., Mininel V.A., Zem-Mascarenhas S.H., Machado R.C. (2019). Strategies to minimize medication errors in emergency units: An integrative review. Rev. Bras. Enferm..

[14] Lincoln E.W., Reed-Schrader E., Jarvis J.L. (2021). EMS Quality Improvement Programs.

[15] Downen J., Jaeger C. (2020). Quality improvement of intravenous to oral medication conversion using Lean Six Sigma methodologies. BMJ Open Qual..

[16] Cutler R.L., Fernandez-Llimos F., Frommer M., Benrimoj C., Garcia-Cardenas V. (2018). Economic impact of medication non-adherence by disease groups: A systematic review. BMJ Open.

[17] Prosci (2018). What Is the ADKAR Model?. http://www.prosci.com/adkar/adkar-model.

[18] Wong Q., Lacombe M., Keller R., Joyce T., O’Malley K. (2019). Leading change with ADKAR. Nurs. Manag..

[19] Kotter J.P. (2012). Accelerate!. Harv. Bus. Rev..

[20] Barrow J.M., Annamaraju P., Toney-Butler T.J. (2021). Change Management.

[21] Kumar A., Nesbitt K.M., Bakkum-Gamez J.N. (2019). Quality improvement in gynecologic oncology: Current successes and future promise. Gynecol Oncol..

